# Transient Receptor Potential Vanilloid 4 Inhibits γ-Aminobutyric Acid-Activated Current in Hippocampal Pyramidal Neurons

**DOI:** 10.3389/fnmol.2016.00077

**Published:** 2016-08-26

**Authors:** Zhiwen Hong, Yujing Tian, Mengwen Qi, Yingchun Li, Yimei Du, Lei Chen, Wentao Liu, Ling Chen

**Affiliations:** ^1^Department of Physiology, Nanjing Medical UniversityNanjing, China; ^2^Research Center of Ion Channelopathy, Institute of Cardiology, Union Hospital, Tongji Medical College, Huazhong University of Science and TechnologyWuhan, China; ^3^Department of Pharmacology, Nanjing Medical UniversityNanjing, China

**Keywords:** TRPV4, GABA_A_ receptor, Ca^2+^, AMPK, PI3K/AKT, phosphorylation

## Abstract

The balance between excitatory and inhibitory neurotransmitter systems is crucial for the modulation of neuronal excitability in the central nervous system (CNS). The activation of transient receptor potential vanilloid 4 (TRPV4) is reported to enhance the response of hippocampal glutamate receptors, but whether the inhibitory neurotransmitter system can be regulated by TRPV4 remains unknown. γ-aminobutyric acid (GABA) is the major inhibitory neurotransmitter in the CNS. Here, we show that application of transient receptor potential vanilloid 4 (TRPV4) synthetic (GSK1016790A or 4α-PDD) or endogenous agonist (5,6-EET) inhibited GABA-activated current (*I*_GABA_) in hippocampal CA1 pyramidal neurons, which was blocked by specific antagonists of TRPV4 and of GABA_A_ receptors. GSK1016790A increased the phosphorylated AMP-activated protein kinase (p-AMPK) and decreased the phosphorylated protein kinase B (p-Akt) protein levels, which was attenuated by removing extracellular calcium or by a calcium/calmodulin-dependent protein kinase kinase-β antagonist. GSK1016790A-induced decrease of p-Akt protein level was sensitive to an AMPK antagonist. GSK1016790A-inhibited *I*_GABA_ was blocked by an AMPK antagonist or a phosphatidyl inositol 3 kinase (PI3K) agonist. GSK1016790A-induced inhibition of *I*_GABA_ was also significantly attenuated by a protein kinase C (PKC) antagonist but was unaffected by protein kinase A or calcium/calmodulin-dependent protein kinase II antagonist. We conclude that activation of TRPV4 inhibits GABA_A_ receptor, which may be mediated by activation of AMPK and subsequent down-regulation of PI3K/Akt signaling and activation of PKC signaling. Inhibition of GABA_A_ receptors may account for the neuronal hyperexcitability caused by TRPV4 activation.

## Introduction

Transient receptor potential vanilloid 4 (TRPV4) is a member of the transient receptor potential superfamily (Benemei et al., 2015). TRPV4 activation induces an inward current that is mainly carried by calcium (Ca^2+^) and helps to depolarize the cell membrane (Garcia-Elias et al., 2014). Activation of TRPV4 increases the spontaneous firing rate in mouse retinal ganglion cells (Ryskamp et al., 2011). In trigeminal ganglion (TG) neurons, the application of a TRPV4 agonist facilitates the production of evoked action potentials (APs; Chen et al., 2009a). In addition to the Ca^2+^ influx through TRPV4 *per se*, TRPV4 activation modulates voltage-gated ion channels and transient receptor potential vanilloid 1 (TRPV1) receptors that are involved in the production or propagation of APs (Liu et al., 2007; Chen et al., 2008a,b, 2009b; Li et al., 2011). In the central nervous system (CNS), activation of TRPV4 by body temperature regulates the resting membrane potential in hippocampal neurons (Shibasaki et al., 2007). Although chronic activation of TRPV4 may increase the expression of the α subunits of voltage-gated sodium channels, acute application of a TRPV4 agonist inhibits the voltage-gated sodium current in hippocampal pyramidal neurons (Hong et al., 2016). In the CNS, the balance between the excitatory and inhibitory neurotransmitter systems is crucial for modulating neuronal excitability. Activation of TRPV4 has been proven to enhance glutamatergic transmission in the hippocampus and to promote glutamate receptor function in hippocampal pyramidal neurons (Cao et al., 2009; Li et al., 2013a,b). However, it remains unclear whether activation of TRPV4 can modulate the inhibitory neurotransmitter system.

γ-aminobutyric acid (GABA) is the major inhibitory neurotransmitter in the adult CNS and acts on three classes of receptors: GABA_A_, GABA_B,_ and GABA_C_ receptors (Sivilotti and Nistri, 1991). GABA_A_ receptors are ligand-gated chloride ion channels that mediate most of the inhibitory activity in the brain (Sivilotti and Nistri, 1991). GABA_A_ receptor inhibition can increase neuronal excitability, and GABA_A_ receptor dysfunction has been implicated in some pathological conditions, including epilepsy, depression, and cerebral ischemic injury (Fritschy and Panzanelli, 2014). It has been reported that acute activation of protease-activated receptor-2 (PAR2) reduces GABA-mediated current in the spinal dorsal horn (Huang et al., 2011). Hyperthermia-induced depression of GABAergic synaptic transmission is observed in the immature rat hippocampus (Qu et al., 2007). As a multiple-activated receptor, TRPV4 is sensitive to mild hyperthermia; in addition, PAR2 can stimulate TRPV4 and sensitize TRPV4-induced currents (Grant et al., 2007). However, there is still a lack of direct evidence for TRPV4-induced modulation of GABA receptors.

GABA_A_ receptor subunits contain phosphorylation sites for protein kinase C (PKC), protein kinase (PKA), Ca^2+^/calmodulin-dependent protein kinase II (CaMKII) and phosphatidyl inositol 3 kinase (PI3K), and these kinases have been reported to be responsible for the TRPV4-induced modulation of some voltage-gated ion channels and glutamate receptors (Chen et al., 2008a, 2009b; Li et al., 2013a; Nakamura et al., 2015). AMP-activated protein kinase (AMPK) can be activated by an increase in Ca^2+^/calmodulin-dependent protein kinase kinase-β (CaMKKβ) activity (Hawley et al., 2005). It has been demonstrated that AMPK can bind directly to and phosphorylate GABA_*B*_ receptors (Kuramoto et al., 2007), but it remains unclear whether activation of AMPK can modulate GABA_A_ receptors. The activation or up-regulation of TRPV1, another member of TRPV family, is accompanied by AMPK phosphorylation (Ching et al., 2012). TRPV4 is an ion channel that is permeable to Ca^2+^; however, it remains to be clarified whether activation of TRPV4 can regulate AMPK signaling. In this study, we first assessed whether GABA-activated current (*I*_GABA_) in hippocampal CA1 pyramidal neurons could be modulated by activation of TRPV4. Then, we examined whether AMPK signaling could be regulated by TRPV4 activation and explored whether AMPK and/or other specific signaling pathways were involved in TRPV4 action.

## Materials and methods

### Experimental animals

Male mice (3-week-old, ICR, Oriental Bio Service Inc., Nanjing, China) were used in this study. All animal procedures used in this study were performed in accordance with the Guidelines for Laboratory Animal Research of Nanjing Medical University and were approved by the Animal Care and Use Committee at Nanjing Medical University. All efforts were made to minimize the animals' suffering and to reduce the number of animals used.

### Slice preparation

The mice were anesthetized with ethyl ether and decapitated, and the brains were rapidly removed. Coronal brain slices (400 μm) were cut using a vibrating microtome (Microslicer DTK 1500, Dousaka EM Co., Kyoto, Japan) in ice-cold modified artificial cerebrospinal fluid (ACSF) containing (in mM) NaCl 126, CaCl_2_1, KCl 2.5, MgCl_2_ 1, NaHCO_3_ 26, KH_2_PO_4_ 1.25, and D-glucose 20. The ACSF was oxygenated with a gas mixture of 95% O_2_/5% CO_2_. The hippocampal slices were incubated in ACSF for 1 h at 32°C to allow them to recover and were then transferred to a recording chamber.

### Whole-cell patch clamp recording

All electrophysiological recordings were performed at room temperature (22–23°C). Hippocampal CA1 pyramidal neurons were viewed with an upright microscope equipped with an infrared-sensitive camera (DAGE-MTI, IR-1000) and in general, the second and the third layer of neurons in the slices were chosen for the patch clamp recording. *I*_GABA_ was recorded using an EPC-10 amplifier (HEKA Elektronik, Lambrecht/Pfalz, Germany) sampled at 10 kHz and filtered (Bessel) at 2.9 kHz. The capacitance and series resistance were compensated (>90%) before recording. Data obtained from neurons in which uncompensated series resistance resulted in voltage-clamp errors >5 mV were not used for subsequent analysis. The liquid junction potentials were compensated before patching.

To record *I*_GABA,_ the holding potential was set at −60 mV. The slices were continually perfused with the oxygenated ACSF containing 0.3 μM TTX. GABA was dissolved in the bath solution and was focally applied using a rapid drug delivery system directed toward the soma of the recorded neurons. Glass pipettes (No. 64-0817 [G85150T-3], Warner Instruments Inc., Hamden, CT, USA) were used with a resistance of 1–3 MΩ when they were filled with the pipette solution composed of (in mM) KCl 140, CaCl_2_ 1, MgCl_2_ 2, EGTA 10, HEPES 10, and Tris-ATP 5 at pH 7.3. The expression of TRPV4 receptors was functionally verified by recording the TRPV4 agonist (GSK1016790A)-activated current as previously reported (Hong et al., 2016).

### Western blot

Western blot analysis was performed at different time points (0, 15, 30 min, 1 and 2 h) after the slices were perfused with GSK1016790A. After the perfusion, the hippocampi were rapidly collected and homogenized in a lysis buffer containing 50 mM Tris-HCl (pH 7.5), 150 mM NaCl, 5 mM EDTA, 10 mM NaF, 1 mM sodium orthovanadate, 1% Triton X-100, 0.5% sodium deoxycholate, 1 mM phenylmethylsulfonyl fluoride, and a protease inhibitor cocktail (Complete; Roche, Mannheim, Germany). Protein concentrations were determined using a bicinchoninic acid (BCA) Protein Assay Kit (Pierce, Rochford, IL, USA). Total proteins were separated by sodium dodecyl sulfate polyacrylamide gel electrophoresis (SDS-PAGE) and were then transferred to a polyvinylidene difluoride (PVDF) membrane. The membranes were incubated with 5% nonfat dried milk in Tris-buffered saline containing 0.1% Tween 20 (TBST) for 60 min at room temperature and were then incubated with an anti-phospho-AMPK antibody (1:1000, Cell Signaling Technology), an anti-AMPK antibody (1:1000, Cell Signaling Technology), an anti-phospho-Akt antibody (1:1000, Cell Signaling Technology), an anti-Akt antibody (1:1000, Cell Signaling Technology), or an anti-glyceraldehyde 3-phosphate dehydrogenase (anti-GAPDH) antibody (1:5000; Abcam) overnight at 4°C. After three washes with TBST, the membranes were incubated with a horseradish peroxidase (HRP)-labeled secondary antibody and then developed using an ECL detection Kit (Amersham Biosciences, Piscataway, NJ). The Western blot bands were scanned and analyzed with image analysis software (NIH). The hippocampal samples obtained from three mice were considered as a set for the Western blot analysis, and the summarized data represent the average of three experimental sets.

### Data analysis

The data are presented as means ± S.E.M. and were analyzed using PulseFit (HEKA Elektronik) and Stata 7.0 software (STATA Corporation, USA). All data came from neurons in which both *I*_GABA_ and GSK1016790A-evoked current could be recorded (Supplementary Figure 1). Paired or unpaired Student's *t*-tests or analysis of variance (ANOVA) followed by Bonferroni's *post hoc* test were used for the statistical analyses, and the significance level was set at either *P* < 0.05 or *P* < 0.01. In the dose-response curve, the *I*_GABA_ induced by different doses of GABA was normalized to the current induced by 300 μM GABA in the same neuron. The data were fitted to a Hill equation in which *I* = *I*_max_/[1+(EC_50_/*C*)^*n*^], with *n* being the Hill coefficient and EC_50_ value being the concentration that produced a 50% maximal response. In the current-voltage relationship curve (I-V curve), *I*_GABA_ induced at different holding potentials was normalized to the current induced with a holding potential of −60 mV in the same neuron.

### Chemicals

5(6)-epoxy-8Z,11Z,14Z-eicosatrienoic acid (5,6-EET) and PKI were obtained from Cayman Chemical (Ann Arbor, MI, USA), and TTX was obtained from Enzo Life Science (Ann Arbor, MI, USA). Unless stated otherwise, all other chemicals were obtained from Sigma Chemical Company. GSK1016790A, 4α-PDD, HC-067047, RN1734, D-sphingosine, bisindolylmaleimide II (BIM), phorbol 12-myristate 13-acetate (PMA), H-89, PKI, 8-bromoadenosine 3′,5′-cyclic monophosphate sodium salt (8-Br-cAMP), LY294002, Compound C, AICAR, STO-609, 740 Y-P, KN62, and KN93 were prepared as stock solutions in DMSO. The final concentration of DMSO in the bath solution or pipette solution was < 0.1%. GSK1016790A, 4α-PDD, 5,6-EET, HC-067047, RN1734, BIM, PMA, 8-Br-cAMP, Compound C, AICAR, STO-609, 740 Y-P, and bicuculline were extracellulary applied by being added to the bath solution and the rapid drug delivery system. When exploring the effect of these chemicals on *I*_GABA_, the slices were pre-incubated by these chemicals. D-Sphingosine, H-89, PKI, LY294002, KN62, and KN93 were present in the pipette solution and pre-applied by dialyzing into the neurons through the pipette. The concentrations of these drugs were chosen according to previous reports (Ben-Ari et al., 1997; Williams and Doherty, 1999; Chen et al., 2000, 2008b; Liu et al., 2007; Langelueddecke et al., 2012; Sisignano et al., 2012; Li et al., 2013a; Shen et al., 2014; Hong et al., 2016; Rahman et al., 2016).

## Results

### Effects of TRPV4 agonists on *I*_GABA_ in hippocampal CA1 pyramidal neurons

**Figure 1:**
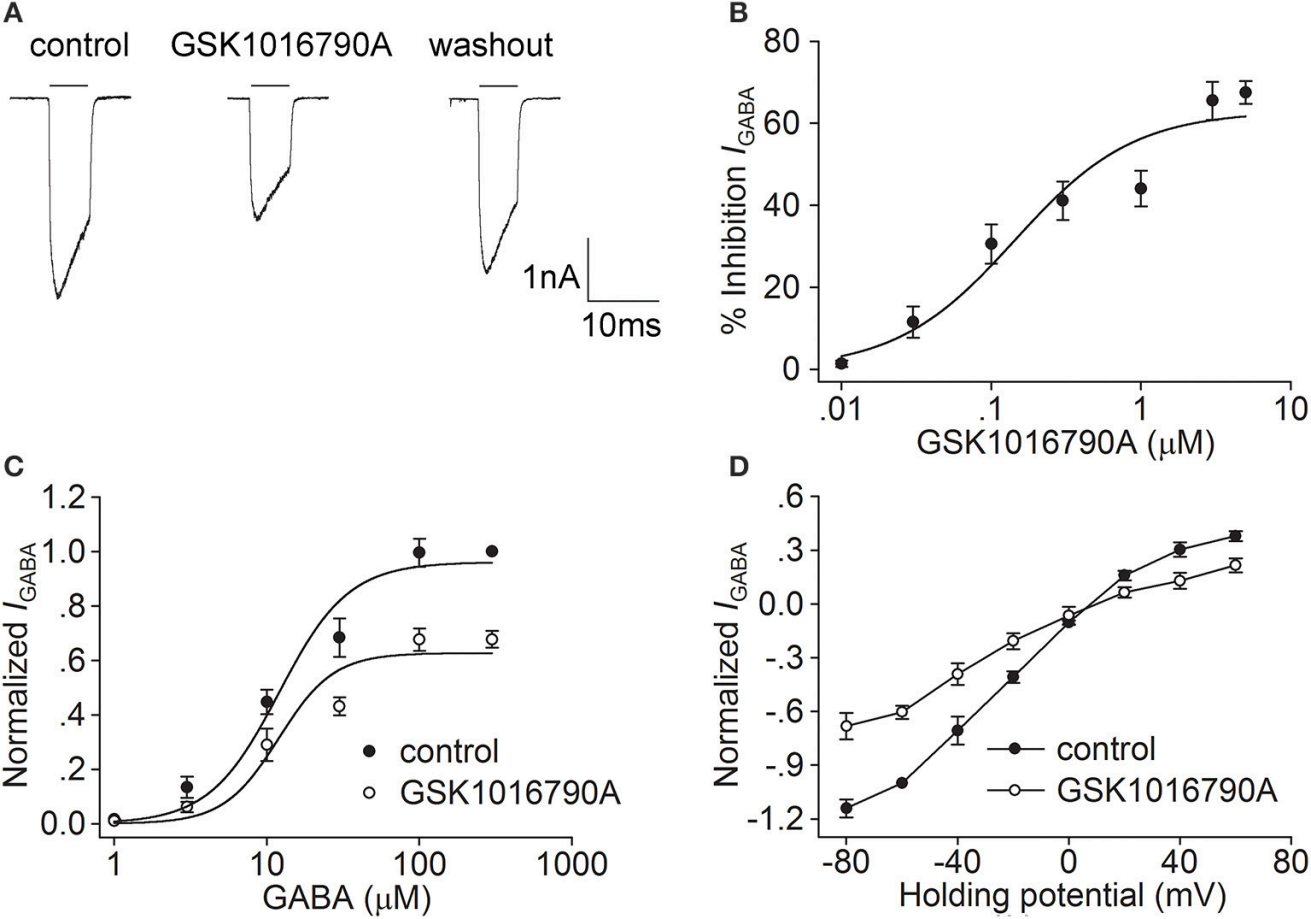
**Effect of GSK1016790A on *I*_GABA_ in hippocampal CA1 pyramidal neurons. (A)** The representative recordings show that *I*_GABA_ (activated by 10 μM GABA) was inhibited from −3.1 to −2.0 nA by 0.3 μM GSK1016790A, and the current recovered to −2.8 nA after washout. **(B)** The plot shows inhibition of *I*_GABA_ by GSK1016790A at concentrations of 0.01, 0.03, 0.1, 0.3, 1, 3, and 5 μM. The dose-response curve fits the Hill equation, with IC_50_value of 0.1 ± 0.05 μM and *n* of 1.09 ± 0.04. **(C)** Dose-response curves for *I*_GABA_ before and during GSK1016790A treatment. Each point represents the normalized current from 8 to 10 neurons. **(D)** I–V curves for *I*_GABA_ before and during GSK1016790A treatment. Each point represents the normalized current from 8 to 10 neurons.

In the present study, the synthetic TRPV4 agonists GSK1016790A and 4α-PDD and the endogenous TRPV4 agonist 5,6-EET were used to determine whether activation of TRPV4 could modulate *I*_GABA_. We found that *I*_GABA_ (activated by 10 μM GABA) was markedly decreased by 41.1 ± 4.7% from −24.4±2.1 to −15.8±3.2 pA/pF after the application of the TRPV4 agonist GSK1016790A (0.3 μM; *n* = 25, paired *t*-test, *P* < 0.01; Figure 1A). The decrease in *I*_GABA_ was partially reversed after GSK1016790A was washed out. The GSK1016790A-induced inhibition of *I*_GABA_ was dose-dependent at concentrations ranging from 0.1 to 5 μM, with an IC_50_ value of 0.1 ± 0.05 μM (Figure 1B). As 0.3 μM GSK1016790A significantly inhibited *I*_GABA_, this dose was used in the subsequent experiments.

We then studied the effect of GSK1016790A on the dose-response of *I*_GABA_. As shown in Figure 1C, in the absence of GSK1016790A, the EC_50_ and *n* values of the dose-response curve were 12.0 ± 2.3 μM and 1.9 ± 0.4, respectively. In the presence of GSK1016790A, the maximal response to 300 μM GABA was markedly decreased (*n* = 7, paired *t*-test, *P* < 0.01), with EC_50_ and *n*-values being 12.0 ± 2.8 μM and 2.4 ± 0.3, respectively (unpaired *t*-test, *P* > 0.05 in each case). According to the dose-response curve, 10 μM GABA was used to activate *I*_GABA_ in the following experiments. We then assessed the effect of GSK1016790A on the I-V curve of *I*_GABA_. *I*_GABA_ was markedly inhibited by the application of GSK1016790A at voltages ranging from −80 mV to +60 mV. In the control group, the reversal potential of the I-V curve was 7.8 ± 0.7 mV, and the ratio of the current at +60/−80 mV (*I*_+60mV_/I_−80mV_) was −0.3. After the neurons were treated with GSK1016790A, the reversal potential of the I-V curve was 8.5 ± 0.5 mV and *I*_+60mV_/I_−80mV_ ratio was −0.3 (*n* = 9, paired *t*-test, *P* > 0.05 in each case; Figure 1D).

**Figure 2:**
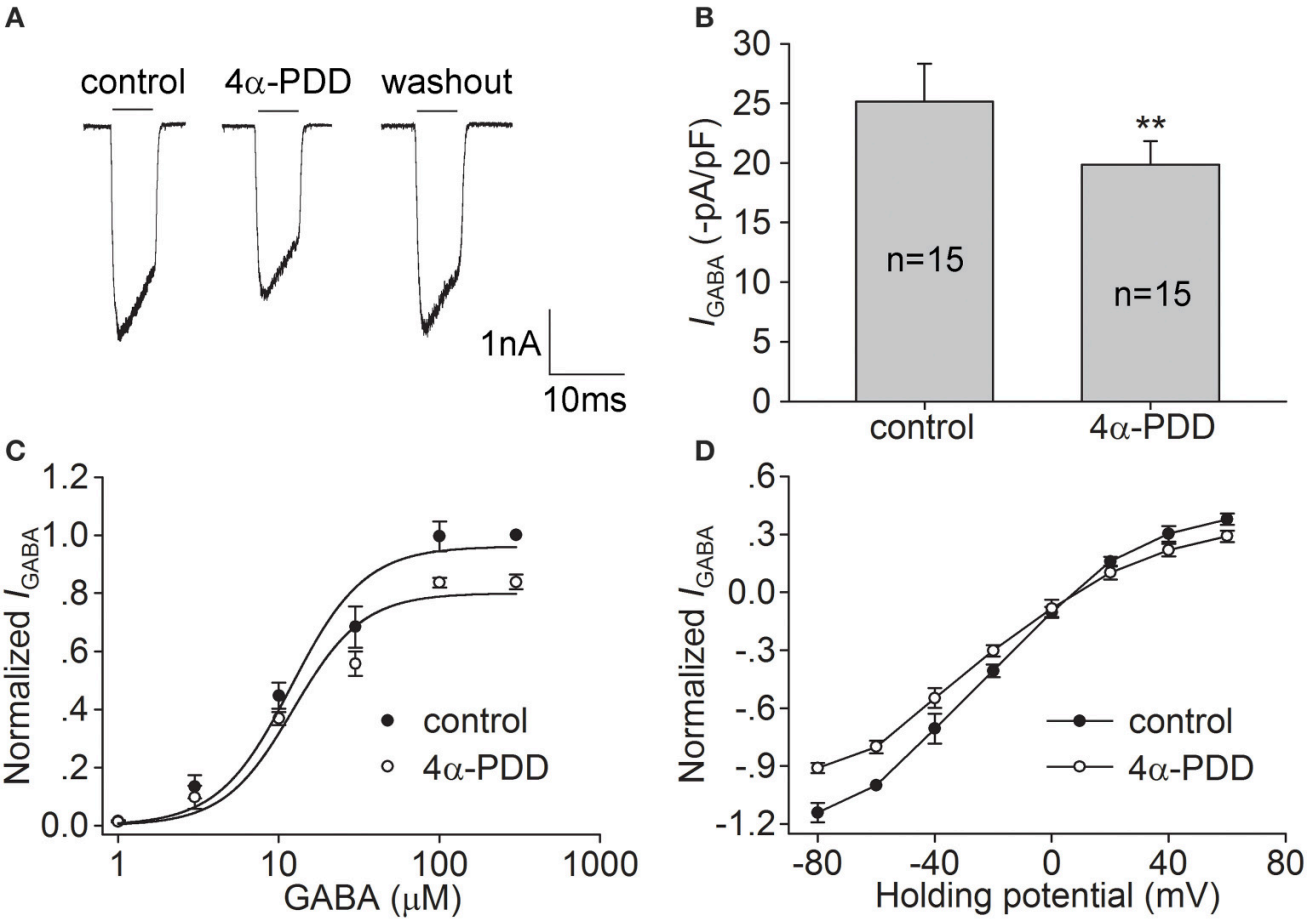
**Effect of 4α-PDD on *I*_GABA_ in hippocampal CA1 pyramidal neurons. (A,B)** *I*_GABA_ (activated by 10 μM GABA) was inhibited by 10 μM 4α-PDD. The representative recordings show that *I*_GABA_ was −3.0, −2.4, and −2.9 nA before, during and after 4α-PDD treatment, respectively **(A)**. On the average, *I*_GABA_ was reduced from −25.2±3.2 to −19.9±1.9 pA/pF. ^**^P < 0.01 vs. control **(B). (C)** Dose-response curves for *I*_GABA_ before and during 4α-PDD treatment. Each point represents the normalized current from 7 to 10 neurons. **(D)** I–V curves for *I*_GABA_ before and during 4α-PDD treatment. Each point represents the normalized current from 7 to 10 neurons.

We then examined the effect of 4α-PDD, another TRPV4 agonist, on *I*_GABA_. As shown in Figures 2A,B, after treatment with 10 μM 4α-PDD, *I*_GABA_ was decreased by 20.0 ± 2.2% from −25.2±3.2 to −19.9±1.9 pA/pF (*n* = 15, paired *t*-test, *P* < 0.01). *I*_GABA_ recovered to −22.6±1.1 pA/pF after 4α-PDD was washed out. By examining the dose-response curve, we found that the EC_50_ and *n*-values were 12.3 ± 2.5 μM and 2.0 ± 0.2 during 4α-PDD treatment, respectively, which were not significantly different from the control values (unpaired *t*-test, *P* > 0.05; Figure 2C). By examining the I-V curve in the presence of 4α-PDD, we found that *I*_GABA_ was inhibited at the voltages ranging from −80 mV to +60 mV (unpaired *t*-test, *P* < 0.01 at potential being −80, −60, −40, −20, and +60 mV; unpaired *t*-test, *P* < 0.05 at potential being +40 mV), with the reversal potential being 8.3 ± 1.0 mV (*n* = 10) and *I*_+60mV_/I_−80mV_ratio being −0.3 (*n* = 10). Neither the reversal potential nor *I*_+60mV_/I_−80mV_ ratio was markedly different from the value in the absence of 4α-PDD (unpaired *t*-test, *P* > 0.05 in each case; Figure 2D). These results indicate that the activation of TRPV4 by the synthetic TRPV4 agonists GSK1016790A and 4α-PDD induces similar inhibitory effects on *I*_GABA_.

**Figure 3:**
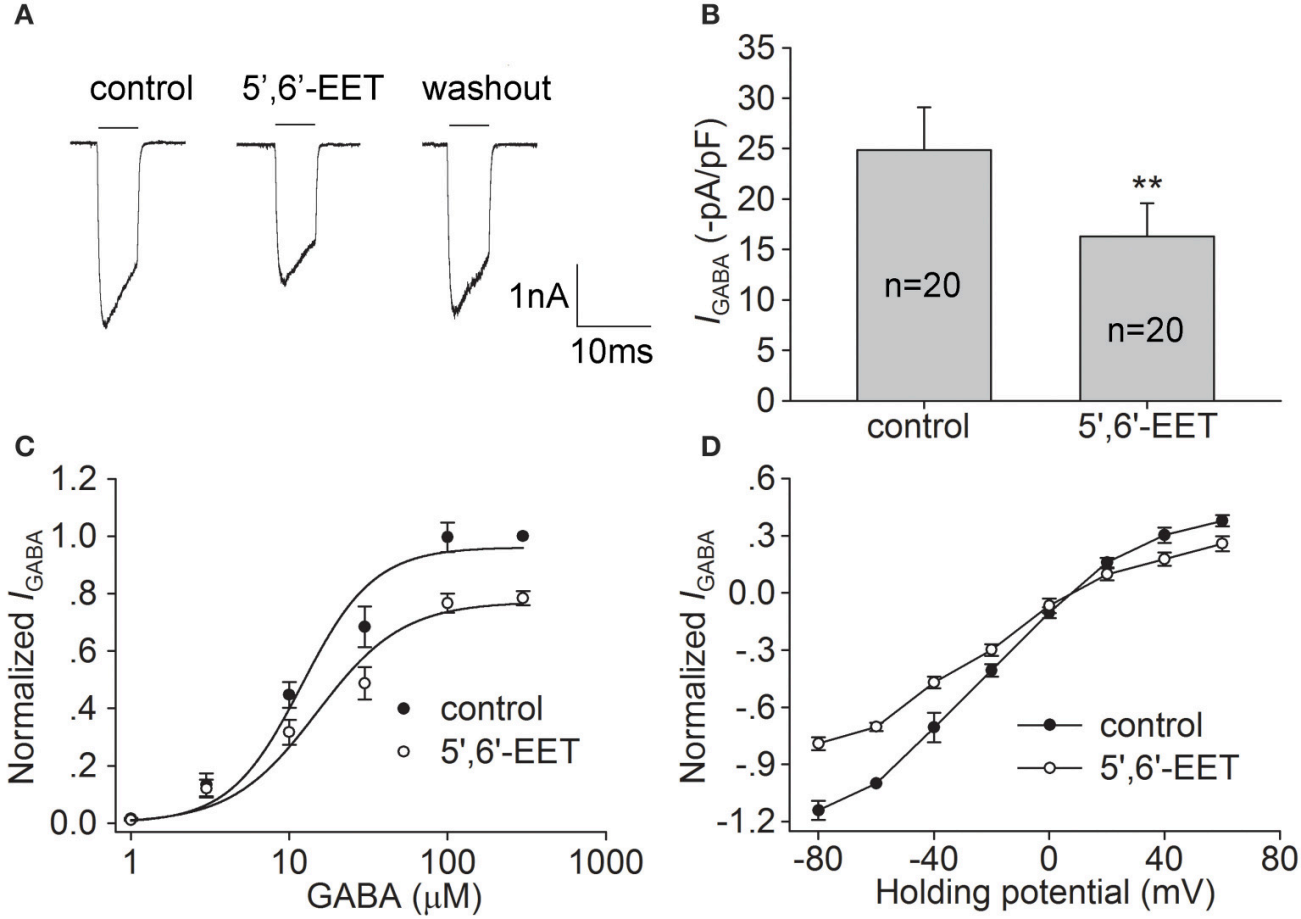
**Effect of 5,6-EET on *I*_GABA_ in hippocampal CA1 pyramidal neurons. (A,B)** *I*_GABA_ (activated by 10 μM GABA) was inhibited by 300 nM 5,6-EET. The representative recordings show that *I*_GABA_ was −3.0, −2.2, and −2.7 nA before, during and after 5,6-EET treatment, respectively **(A)**. On the average, *I*_GABA_ was reduced from −24.9±4.2 to −16.3±3.3 pA/pF. ^**^P < 0.01 vs. control **(B). (C)** Dose-response curves for *I*_GABA_ before and during 5,6-EET treatment. Each point represents the normalized current from 8 to 10 neurons. **(D)** I–V curves for *I*_GABA_ in the absence and presence of 5,6-EET. Each point represents the normalized current from 8 to 10 neurons.

5,6-EET is a metabolite of arachidonate and has been identified as an endogenous TRPV4 agonist (Vincent and Duncton, 2011). In the present study, we also examined the effect of 5,6-EET on *I*_GABA_. Figures 3A,B show that after treatment with 300 nM 5,6-EET, *I*_GABA_ was decreased by 28.1 ± 4.9% from −24.9±4.2 to −16.3±3.3 pA/pF (*n* = 20, paired *t*-test, *P* < 0.01), and the inhibitory effect of 5,6-EET on *I*_GABA_ was partially reversed after washout. As shown in Figure 3C, similar to the effect of the synthetic agonists of TRPV4, 5,6-EET inhibited the maximal response to 300 μM GABA (*n* = 17, paired *t*-test, *P* < 0.01), without affecting the EC_50_ (15.0 ± 3.1 μM) or *n* (2.1 ± 0.1) value of the dose-response curve (unpaired *t*-test, *P* > 0.05 in each case). In the I-V curve, the reversal potential (8.0 ± 0.8 mV, *n* = 8) and *I*_+60mV_/I_−80mV_ ratio (−0.3, *n* = 8) were statistically the same as the values before the 5′6′-EET treatment (unpaired *t*-test, *P* > 0.05 in each case; Figure 3D). These results indicate that activation of TRPV4 by either synthetic or endogenous agonists could inhibit *I*_GABA_.

### Effects of HC-067047, RN1734, and bicuculline on TRPV4 agonist-induced inhibition of *I*_GABA_

**Figure 4:**
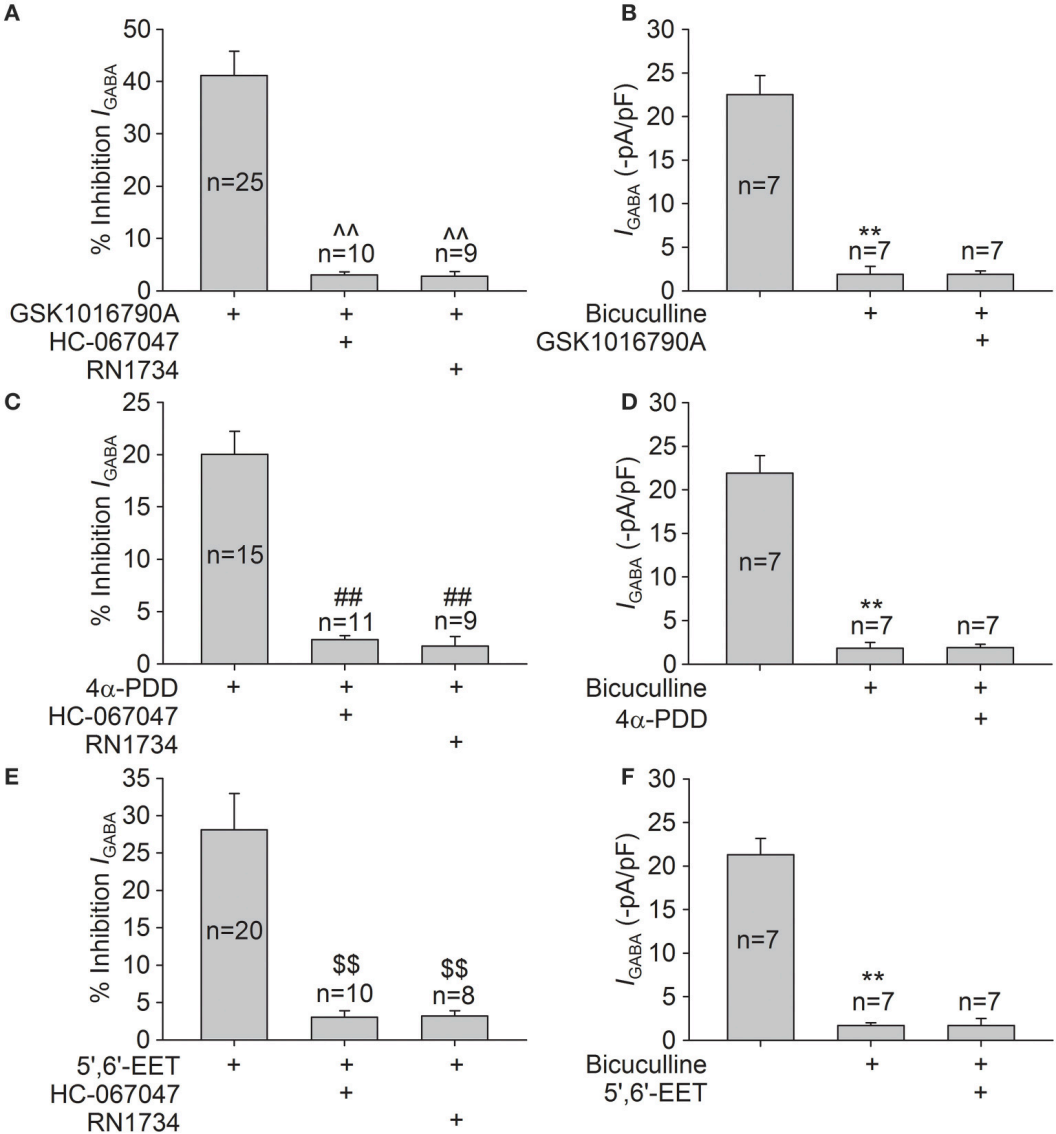
**Effects of HC-067047, RN1734, and bicuculline on TRPV4 agonists-induced inhibition of *I*_GABA_. (A)** In the presence of HC-067047 or RN1734, the inhibition of *I*_GABA_ by GSK1016790A was decreased from 41.1 ± 4.7% (*n* = 25) to 3.0 ± 0.6% (*n* = 10) or to 2.8 ± 0.9% (*n* = 9), respectively. Unpaired *t*-test, ^∧∧^*P* < 0.01 vs. GSK1016790A. **(B)** *I*_GABA_ was markedly inhibited from −22.5±2.2 to −1.9±0.9 pA/pF by the application of bicuculline, and the current was virtually unaffected by GSK1016790A treatment (−1.9±0.4 pA/pF) in the presence of bicuculline. Paired *t*-test, ^**^P < 0.01 vs. control, *n* = 7. **(C)** 4α-PDD-induced inhibition of *I*_GABA_ was significantly attenuated from 20.0 ± 2.2% (*n* = 15) to 2.3 ± 0.4% (*n* = 11) or to 1.7 ± 0.9% (*n* = 9) by pre-application of HC-067047 or RN1734, respectively. Unpaired *t*-test, ^*##*^*P* < 0.01 vs. 4α-PDD. **(D)** In the presence of bicuculline, the current (−1.8±0.7 pA/pF) was virtually unchanged by 4α-PDD treatment (−1.9±0.4 pA/pF). Paired *t*-test, ^**^P < 0.01 vs. control, *n* = 7. **(E)** After pre-application of HC-067047 or RN1734, the inhibition of *I*_GABA_ by 5,6-EET was reduced from 28.1 ± 4.9% (*n* = 20) to 3.0 ± 0.9% (*n* = 10) or to 3.2 ± 0.7% (*n* = 8), respectively. Unpaired *t*-test, $$*P* < 0.01 vs. 5,6-EET. **(F)** In the presence of bicuculline, the currents were −1.7±0.3 and −1.7±0.8 pA/pF before and during 5,6-EET treatment, respectively. Paired *t*-test, ^**^P < 0.01 vs. control, *n* = 7.

Specific TRPV4 antagonists, HC-067047 (1 μM) and RN1734 (10 μM), were used to further demonstrate the role of TRPV4 in *I*_GABA_ inhibition. *I*_GABA_ was −22.7±2.4 and −20.5±2.7 pA/pF before and during HC-067047 treatment, respectively (*n* = 8, paired *t*-test, *P* > 0.05). *I*_GABA_ was −23.2±1.0 and −21.7±1.3 pA/pF in the absence and presence of RN1734, respectively (*n* = 7, paired *t*-test, *P* > 0.05). As shown in Figure 4, in the presence of HC-067047 or RN1734, the inhibition caused by GSKA1016790, 4α-PDD, or 5,6-EET was markedly ameliorated (unpaired *t*-test, *P* < 0.01 in each case; Figures 4A,C,E).

Application of bicuculline (10 μM), a specific GABA_A_ receptor antagonist, markedly reduced *I*_GABA_ by 93.6 ± 1.8% (*n* = 21, paired *t*-test, *P* < 0.01). In the presence of bicuculline, *I*_GABA_ was statistically the same before and during treatment with TRPV4 agonists (Figures 4B,D,F). Together, these results imply that GABA_A_ receptor is inhibited by activation of TRPV4.

### Involvement of intracellular signaling pathways in GSK1016790A-induced inhibition of *I*_GABA_

**Figure 5:**
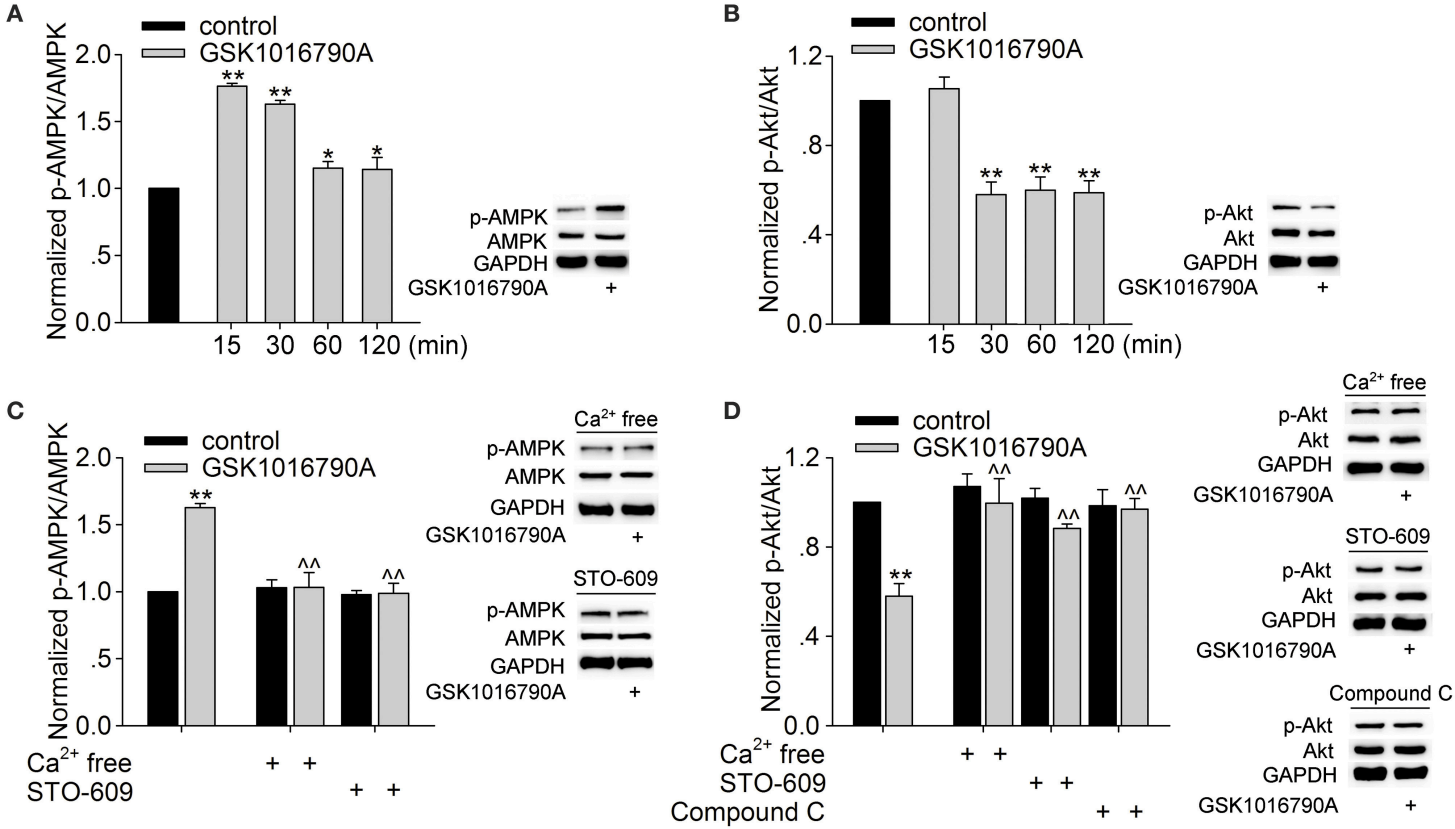
**Effect of GSK1016790A on p-AMPK and p-Akt levels**. **(A,B)** Western blot analysis of p-AMPK **(A)** and p-Akt **(B)** levels in the hippocampus 15, 30 min, 1 and 2 h after GSK1016790A treatment. The p-AMPK or p-Akt protein density was first normalized to the density of AMPK or Akt, respectively, and the densities of the GSK1016790A-treated group were then normalized to the control values at each of the different time points. Each group contained 9 mice. ^**^P < 0.01 vs. control. **(C)** Western blot analysis of p-AMPK protein levels in the hippocampus 30 min after GSK1016790A treatment. The increase in the p-AMPK protein level was blocked when the extracellular Ca^2+^ was removed or when the slices were pre-incubated with the CaMKKβ antagonist STO-609. ^**^P < 0.01 vs. control, ^∧∧^*P* < 0.01 vs. GSK1016790A. Each group contained 9 mice. **(D)** Western blot analysis of p-Akt protein levels in the hippocampus 30 min after GSK1016790A treatment. The decrease in the p-Akt protein level was markedly attenuated when the extracellular Ca^2+^ was removed or when the slices were pre-incubated with the AMPK antagonist Compound C or the CaMKKβ antagonist STO-609. ^*^P < 0.05, ^**^P < 0.01 vs. control, ^∧∧^*P* < 0.01 vs. GSK1016790A. Each group contained 9 mice.

The cellular energy-sensing kinase AMPK is known to be activated in neurons in response to either metabolic insults or increased [Ca^2+^]_i_ through CaMKKβ. Activation of AMPK is related to modulating PI3K/Akt signaling, and the latter is involved in the modulation of GABA_A_ receptors (Amato et al., 2011; Nakamura et al., 2015). As TRPV4 is permeable to Ca^2+^, we examined whether activation of TRPV4 could affect AMPK-PI3K/Akt signaling. The protein levels of phosphorylated AMPK (p-AMPK) and phosphorylated Akt (p-Akt) in the hippocampi were assessed after the slices were perfused with ACSF containing GSK1016790A for 15, 30 min, 1 and 2 h. The protein level of p-AMPK was increased 15 min to 2 h after GSK1016790A treatment; the level peaked 15 min after GSK1016790A treatment and then declined (Figure 5A). The protein level of p-Akt decreased from 30 min to 2 h after GSK1016790A treatment (Figure 5B). Based on the changes in the levels of p-AMPK and p-Akt and the acute effect of TRPV4 activation, the changes in protein levels were examined 30 min after GSK1016790A treatment in the subsequent experiments. Here, it was noted that both the GSK1016790A-mediated increase in the protein level of p-AMPK and the GSK1016790A-mediated decrease in the protein level of p-Akt were markedly attenuated when the slices were perfused with Ca^2+^-free ACSF. In the presence of 10 μM STO-609, a CaMKKβ antagonist, the GSK1016790A-induced changes in the protein levels of p-AMPK, and p-Akt were markedly inhibited (Figures 5C,D). The GSK1016790A-induced decrease in the protein level of p-Akt was significantly reversed by the application of 10 μM Compound C, an AMPK antagonist (Figure 5D). These results indicate that TRPV4-induced activation of AMPK is Ca^2+^- and CaMKKβ-dependent and this action then down-regulates Akt signaling.

**Figure 6:**
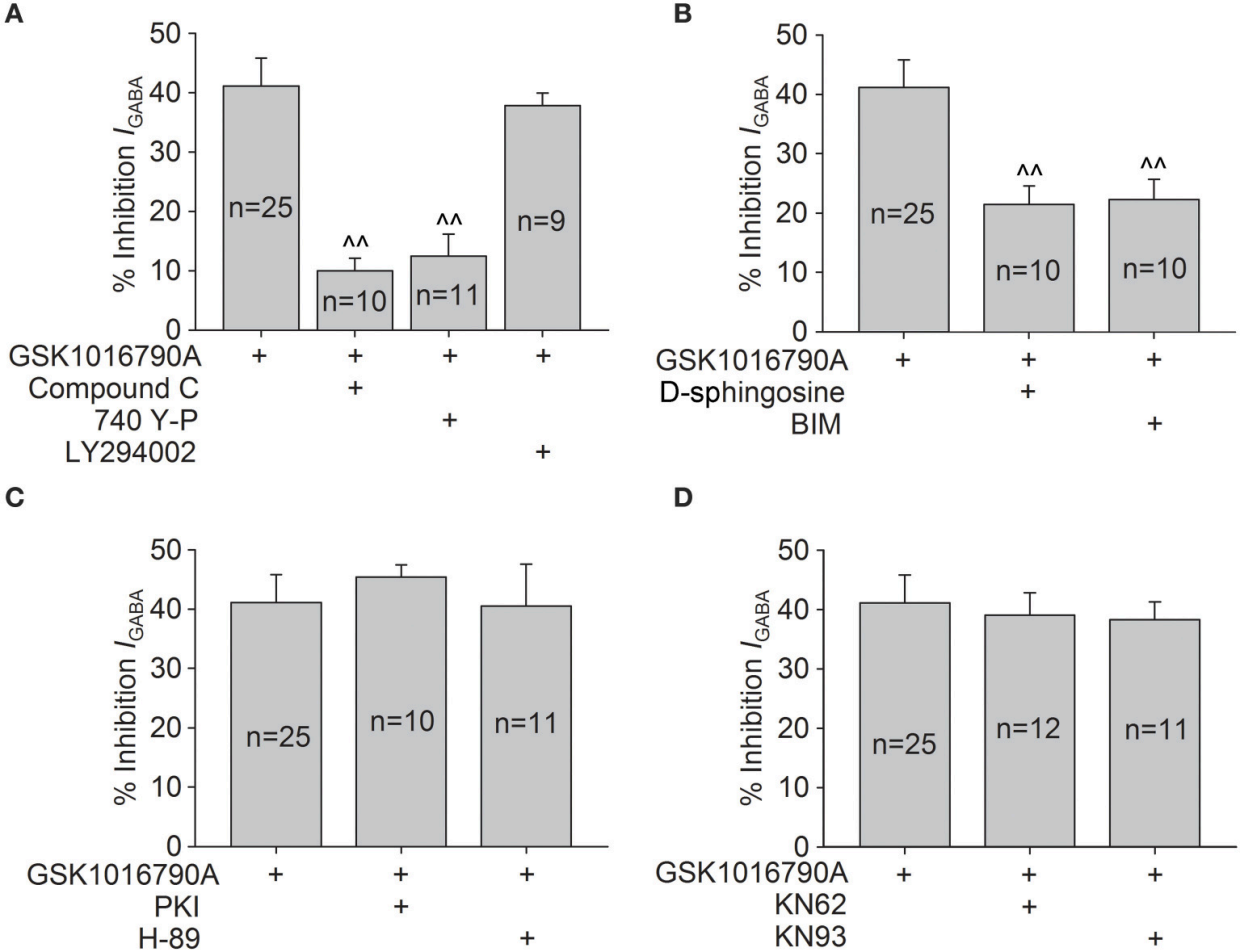
**Intracellular signaling pathways involved in GSK1016790A-induced inhibition of *I*_GABA_**. **(A)** GSK1016790A-induced inhibition of *I*_GABA_ was markedly attenuated by either the AMPK antagonist Compound C or the PI3K agonist 740 Y-P but was not affected by the PI3K antagonist LY294002. Unpaired *t*-test, ^∧∧^*P* < 0.01 vs. GSK1016790A. **(B)** In the presence of the PKC antagonist D-sphingosine or BIM, the GSK1016790A-induced inhibition of *I*_GABA_ was significantly reversed. Unpaired *t*-test, ^∧∧^*P* < 0.01 vs. GSK1016790A. **(C,D)** Pre-application of the PKA antagonists PKI or H-89 **(C)** or the CaMKII antagonists KN62 or KN93 **(D)** did not affect the GSK1016790A-induced inhibition of *I*_GABA_.

We then evaluated whether the regulation of AMPK and Akt signaling was involved in TRPV4-induced inhibition of *I*_GABA_. *I*_GABA_ was reduced by 20.55 ± 2.57% when 1 mM AICAR, an AMPK agonist, was applied to the bath solution (*n* = 18, paired *t*-test, *P* < 0.01). We also found that *I*_GABA_ was increased by 11.84 ± 3.15% when the slices were exposed to Compound C (*n* = 10, paired *t*-test, *P* < 0.05). These results indicate that activation of AMPK plays a role in the regulation of *I*_GABA_. As shown in Figure 6A, after pre-application of Compound C, GSK1016790A reduced *I*_GABA_ by 10.0 ± 2.1%, which was markedly different from the inhibition caused by GSK1016790A alone (unpaired *t*-test, *P* < 0.01).

In this study, *I*_GABA_ was increased 19.1 ± 3.1% by the application of 20 μM 740 Y-P (a PI3K agonist; *n* = 11, paired *t*-test, *P* < 0.01) and was inhibited 22.3 ± 4.2% by the application of 50 μM LY294002 (a PI3K antagonist; *n* = 7, paired *t*-test, *P* < 0.01). In the presence of 740 Y-P, GSK1016790A-induced inhibition of *I*_GABA_ was reduced to 12.5 ± 3.6% (*n* = 11, paired *t*-test, *P* < 0.01); however, the GSK1016790A-induced inhibition of *I*_GABA_ was virtually unaltered (37.8 ± 2.1%; *n* = 9) by the application of LY294002 (unpaired *t*-test, *P* > 0.05; Figure 6A). Collectively, these results indicate that the activation of AMPK and the subsequent down-regulation of the PI3K/Akt signaling pathway are likely responsible for the inhibition of *I*_GABA_ caused by acute activation of TRPV4.

We also investigated whether PKC, PKA, or CaMKII signaling was involved in TRPV4-induced inhibition of *I*_GABA_. After the application of PKC antagonist D-sphingosine (20 μM) or BIM (1 μM), *I*_GABA_ was inhibited by 21.5 ± 3.1% (*n* = 10) or 22.3 ± 3.4% (*n* = 10) by GSK1016790A, which was significantly different from the inhibition induced by GSK1016790A alone (unpaired *t*-test, *P* < 0.01; Figure 6B). As shown in Figure 6C, in the presence of the PKA antagonist PKI (10 μM) or H-89 (10 μM), GSK1016790A treatment inhibited *I*_GABA_ by 45.4 ± 2.0% (*n* = 10) or 40.5 ± 3.1% (*n* = 11), respectively. Both inhibition levels were similar to the inhibition caused by GSK1016790A alone (unpaired *t*-test, *P* > 0.05). Figure 6D shows that when CaMKII antagonist KN62 or KN93 was added in the pipette solution, GSK1016790A inhibited *I*_GABA_ by 39.1 ± 3.7% (*n* = 12) or 38.3 ± 3.0% (*n* = 11), respectively. These results indicate that in addition to AMPK-PI3K/Akt signaling, the PKC signaling pathway is also involved in GSK1016790A-induced inhibition of *I*_GABA_.

## Discussion

GABA is the principal inhibitory neurotransmitter in the mammalian brain. By now, three classes of GABA receptors have been identified in the CNS and GABA_A_ receptors are the main type of ionotropic GABA receptor (Sivilotti and Nistri, 1991; Fritschy and Panzanelli, 2014). Changes in the expression or function of GABA_A_ receptors are important for the modulation of CNS function. Although, there are reports demonstrating that hyperthermia and PAR2, two factors that are related to the activation of TRPV4, may negatively regulate GABA-mediated inhibitory post-synaptic currents, there is still a lack of direct evidence for the TRPV4-induced modulation of GABA_A_ receptors (Qu et al., 2007; Huang et al., 2011). In the present study, *I*_GABA_ recorded in hippocampal CA1 pyramidal neurons was largely blocked by bicuculline (Figures 4B,D,F), indicating that the current was mediated by GABA_A_ receptors. *I*_GABA_ was inhibited by the application of two types of TRPV4 agonist, i.e., the specific synthetic agonists GSK1016790A and 4α-PDD and the endogenous agonist 5,6-EET (Figures 1, 2, 3). Moreover, the inhibition of *I*_GABA_ induced by GSK1016790A, 4α-PDD or 5,6-EET was almost completely blocked by the TRPV4 specific antagonists HC-067047 and RN1734 (Figures 4A,C,E). Therefore, our data provide the first direct evidence that GABA_A_ receptor can be inhibited by acute activation of TRPV4. The subsequent experiments showed that the EC_50_ values in the dose-response curves of GABA_A_ receptor were unaffected by GSK1016790A, 4α-PDD, or 5,6-EET (Figures 1C, 2C, 3C), indicating that TRPV4-induced inhibition of *I*_GABA_ is noncompetitive and is likely not due to decreasing ligand-binding affinity. By assessing the I–V curves, we showed that the reversal voltage and *I*_+60mV_/*I*_−80mV_ ratio were not markedly affected by TRPV4 agonists (Figures 1D, 2D, 3D), suggesting that TRPV4 acts in a voltage-independent manner.

GABA_A_ receptors contain phosphorylation sites for protein kinases and phosphorylation plays an important role in the modulation of many aspects of the receptor, including directly regulating channel function and receptor trafficking (Nakamura et al., 2015). AMPK is a heterotrimeric serine/threonine protein kinase and there is evidence that AMPK can be activated by CaMKKβ in a manner that is dependent on an increase in intracellular Ca^2+^ (Hawley et al., 2005). The activation of TRPV1, another TRPV subfamily member, elevates the intracellular Ca^2+^ level. Studies using vascular smooth muscle cells, endothelial cells, and ventricular tissue have demonstrated that the AMPK signaling pathway can be activated by TRPV1-induced elevation in cytosolic Ca^2+^ level (Ching et al., 2012; Lu and Xu, 2013; Li et al., 2014). In addition, the activation of transient receptor potential canonical (TRPC), a member of the TRP superfamily that is also permeable to Ca^2+^, leads to activation of AMPK in CT-26 murine colon cancer cells and human endothelial cells (Bair et al., 2009; Hwang et al., 2013). As TRPV4 acts as a Ca^2+^ channel, we proposed that activation of TRPV4 might activate AMPK signaling pathway. This proposal was confirmed by our data that p-AMPK protein levels were markedly increased in response to GSK1016790A treatment. Moreover, GSK1016790A-increased p-AMPK protein level was blocked if extracellular Ca^2+^ was removed or if the slices were pre-incubated with STO-609, a CaMKKβ inhibitor (Figures 5A,C). These results indicate that activation of TRPV4 may increase AMPK signaling in a manner that is dependent on both Ca^2+^ influx and CaMKKβ. AMPK signaling has been reported to inhibit the PI3K/Akt pathway (Amato et al., 2011). Here, along with the increased AMPK activation, the decrease of p-Akt protein level was significant 30 min to 2 h after GSK1016790A treatment (Figure 5B) and the GSK1016790A-action was reversed by either an AMPK antagonist (Compound C) or a CaMKKβ antagonist (STO-609) (Figure 5D). Therefore, it is likely that activation of TRPV4 activates AMPK and then down-regulates PI3K/Akt signaling. The activation of the PI3K/Akt signaling pathway leads to an increase in GABA_A_ receptor expression on the surfaces of many types of cells, including neurons, α islet cells, and HEK293 cells and is responsible for the potentiation of GABAergic synaptic transmission (Wang et al., 2003; Xu et al., 2006; Guimond et al., 2014). Consistently, the present result showed that *I*_GABA_ was increased by activation of PI3K. Here, it is noted that that the GSK1016790A-induced inhibition of *I*_GABA_ was markedly blocked by pre-application of a PI3K agonist or an AMPK antagonist (Figure 6A). Collectively, our results suggest that activation of AMPK and the subsequent down-regulation of PI3K/Akt signaling are responsible for TRPV4-induced inhibition of *I*_GABA_.

A number of studies have reported that GABA_A_ receptors can be modulated by PKA-, PKC,- and CaMKII-dependent phosphorylation (Nakamura et al., 2015). Studies performed on trigeminal ganglion neurons and hippocampal pyramidal neurons have reported that these signaling pathways are involved in the TRPV4-induced regulation of voltage-gated sodium and potassium currents and N-methyl-D-aspartate (NMDA)-activated currents (Chen et al., 2008a, 2009b; Li et al., 2013a). Here, we also explored which, if any, of these kinases are involved in the effect of GSK1016790A on *I*_GABA_. We first determined that *I*_GABA_ was decreased by either activation of the PKC and PKA signaling pathways or inhibition of CaMKII (Supplementary Table 1). The following experiment showed that GSK1016790A-induced inhibition of *I*_GABA_ was markedly blocked by pre-application of a PKC antagonist (BIM or D-Sphingosine) but was unaffected by either PKA or CaMKII antagonists (Figures 6B–D). Therefore, in addition to AMPK-PI3K/Akt signaling, PKC signaling pathway is also involved in GSK1016790A-induced inhibition of *I*_GABA_. It is known that PKC can modulate GABA_A_ receptors by changing the channel conductance or altering GABA_A_ receptor trafficking (Song and Messing, 2005). Activation of the PI3K/Akt pathway has been shown to increase the number of GABA_A_ receptors on the membrane surface, which is due to a rapid translocation of intracellular receptors to the plasma membrane (Wang et al., 2003). Therefore, it was proposed that TRPV4-induced inhibition of *I*_GABA_ probably results from a direct decrease in GABA_A_ receptor conductance and/or the total number of GABA_A_ receptors on the cell surface and additional experiments are required to prove this hypothesis.

The GABAergic system is of great importance in regulating neuronal excitability and network oscillation dynamics and thus, plays a crucial role in brain function. In hippocampal dentate gyrus, activation of TRPV1 has been proven to inhibit somatic GABAergic synaptic function through promoting internalization of GABA_A_ receptor (Chávez et al., 2014). This study shows that TRPV4 activation may inhibit GABA_A_ receptor and thus provides a possibility that activation of TRPV4 may negatively regulate GABAergic synaptic function. More experiments are needed to clarify this through assessing the evoked and miniature inhibitory postsynaptic current. Glutamatergic synaptic transmission and the function of glutamate receptors [including NMDA and α-amino-3-hydroxy-5-methl-4-isoxazolepropionic acid (AMPA) receptors] can be enhanced by TRPV4 activation. Here, inhibition of GABA_A_ receptors may further aggravate the imbalance between the excitatory and inhibitory systems and thereby helps to account for the increased neuronal excitability caused by TRPV4 activation. Another important finding of this study was that we demonstrated, for the first time, that AMPK-PI3K/Akt signaling was responsible for regulating *I*_GABA_, which provides new insights into the modulation of GABA_A_ receptors.

## Author contributions

ZH, YT, and MQ performed experiments; YL and YD analyzed data; Lei Chen and WL designed experiments; Lei Chen wrote the article; YD and Ling Chen revised the manuscript and all authors approved the final version.

### Conflict of interest statement

The authors declare that the research was conducted in the absence of any commercial or financial relationships that could be construed as a potential conflict of interest.
