# Supplementary material for: Transient Receptor Potential Vanilloid 4 Inhibits γ-Aminobutyric Acid-Activated Current in Hippocampal Pyramidal Neurons
Source: Front Mol Neurosci. 2016 Aug 26;9:77. doi: 10.3389/fnmol.2016.00077 (PMC4999446; doi:10.3389/fnmol.2016.00077)
Supplement: Supplementary file 1 [file Table1.PDF]

**Supplementary Table 1 Effect of intracellular signaling systems on $I_{GABA}$**

| Intracellular signaling system | | $I_{GABA}$ (pA/pF) | | |
|---|---|---|---|---|
| | | control | agonist/antagonsit | n |
| AMPK | AICAR (1 mM) | -24.53±2.42 | -19.84±1.74** | 18 |
| | Compound C (10 μM) | -23.13±1.14 | -25.68±2.13* | 10 |
| PI3K | 740 Y-P (20 μM) | -24.61±3.51 | -30.34±3.10** | 14 |
| | LY294002 (50 μM) | -25.04±2.24 | -19.58±3.30** | 8 |
| PKC | PMA(1 μM) | -25.08±2.66 | -29.08±1.32** | 10 |
| | D-sphingosine (20 μM) | -24.78±2.50 | -29.77±2.09* | 8 |
| | BIM (1 μM) | -25.14±1.97 | -28.98±2.09** | 12 |
| PKA | 8-Br-cAMP (1mM) | -24.22±2.80 | -19.26±2.63** | 16 |
| | PKI (10 μM) | -24.89±3.07 | -27.79±1.07* | 10 |
| | H-89 (10 μM) | -25.17±2.09 | -28.62±1.11* | 9 |
| CaMKII | KN62 (5 μM) | -25.37±3.41 | -20.80±1.85** | 10 |
| | KN93 (5 μM) | -24.98±2.73 | -19.05±1.46** | 9 |

Paired t test was used to evaluate the effect of agonists or antagonists on $I_{GABA}$. *$P$<0.05, **$P$<0.01 vs. control.
