# Supplementary material for: Transient Receptor Potential Vanilloid 4 Inhibits γ-Aminobutyric Acid-Activated Current in Hippocampal Pyramidal Neurons
Source: Front Mol Neurosci. 2016 Aug 26;9:77. doi: 10.3389/fnmol.2016.00077 (PMC4999446; doi:10.3389/fnmol.2016.00077)
Supplement: Supplementary file 2 [file Presentation1.PDF]

Supplementary Figure legend

Figure 1 I_{GABA} and the GSK1016790A-induced current recorded in hippocampal CA1 pyramidal neurons

When recording the TRPV4-mediated current, 500 nM GSK1016790A and 0.3 μM TTX were added to the ACSF, and a ramp protocol that depolarized from −80 mV to +80 mV over 700 ms was used. The representative recordings show that I_{GABA} (*left*) and the GSK1016790A-induced current (*right*) were recorded in the same neuron.
