# Supplementary figures and images for: Transient Receptor Potential Vanilloid 4 Inhibits γ-Aminobutyric Acid-Activated Current in Hippocampal Pyramidal Neurons
Source: Front Mol Neurosci. 2016 Aug 26;9:77. doi: 10.3389/fnmol.2016.00077 (PMC4999446; doi:10.3389/fnmol.2016.00077)

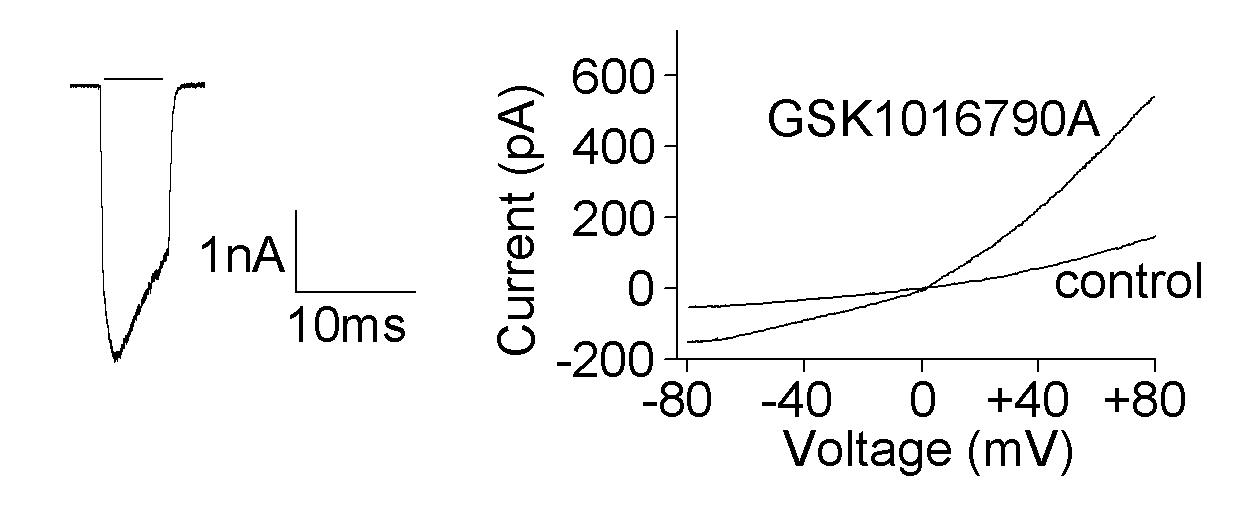

Supplement: Supplementary file 3 [file Image1.TIF]
